# Decomposing Disability Inequality in Unmet Healthcare Needs and Preventable Hospitalizations: An Analysis of the Korea Health Panel

**DOI:** 10.3389/ijph.2023.1605312

**Published:** 2023-02-28

**Authors:** Sujin Kim, Boyoung Jeon

**Affiliations:** ^1^ Korea Institute for Health and Social Affairs, Sejong, Republic of Korea; ^2^ Department of Health and Medical Information, Myongji College, Seoul, Republic of Korea

**Keywords:** disability, unmet healthcare needs, healthcare disparity, preventable hospitalization, decomposition analysis

## Abstract

**Objectives:** This study examines the inequality between people with and without disabilities regarding unmet healthcare needs and preventable hospitalization.

**Methods:** We used the Korea Health Panel of 2016–2018; the final analytical observations were 43,512, including 6.95% of persons with disabilities. We examined the differences in contributors to the two dependent variables and decomposed the observed differences into explained and unexplained components using the Oaxaca-Blinder approach.

**Results:** Unmet healthcare needs and preventable hospitalizations were 5.6% p (15.36% vs. 9.76%) and 0.68% p (1.82% vs. 0.61%), respectively, higher in people with disabilities than in those without, of which 48% and 35% were due to characteristics that the individual variables cannot explain. Decomposition of the distributional effect showed that sex, age, and chronic disease significantly increased disparities for unmet healthcare needs and preventable hospitalization. Socioeconomic factors such as income level and Medical aid significantly increased the disabled–non-disabled disparities for unmet healthcare needs.

**Conclusion:** Socioeconomic conditions increased the disparities, but around 35%–48% of the disparities in unmet healthcare needs and preventable hospitalization were due to unexplained factors, such as environmental barriers.

## Introduction

One billion people, 15% of the world’s population, experience some form of disability (1). Disability-inclusive development is increasing globally; for example, the United Nations Convention on the Rights of Persons with Disabilities promotes the full integration of persons with disabilities in societies. Additionally, the 2030 Agenda for Sustainable Development clearly states that disability cannot be a reason for the lack of access to development programming or human rights (1). In this regard, there have been many efforts to achieve “better health for all people with disabilities” worldwide; the WHO global disability action plan seeks to remove barriers and improve access to health services and programs as one of their objectives between 2014 and 2021 (2). The health targets for people with disabilities were included in *Healthy People 2000* and later expanded in *Healthy People 2010* and *Healthy People 2020* (3). However, little research has investigated disability disparities within the broader health disparities field; there are calls to reduce the disparities and include people with disabilities in the research area (3, 4).

People with disabilities have a higher prevalence of chronic diseases and are less likely to receive preventive care than persons without disabilities (5). Mainly, people with multiple types of limitations are more likely to have problems receiving clinical preventive services, such as dental checkups and cancer screenings (6), and have poor health outcomes, such as chronic conditions and health status (7).

In Korea, the government established a national registration system for the disabled population according to the “Welfare of People with Disability Act” in 1988. Based on the system, the government provides welfare benefits for people with disabilities according to the level of legal disability, including 15 types of disabilities-physical disabilities, brain lesion disorders, visual impairment, hearing impairment, language disabilities, intellectual disabilities, autistic disorders, mental disabilities, renal impairment, cardiac impairment, respiratory impairment, hepatic impairment, facial disfigurement, intestinal or urinary fistula, and epilepsy disorder (8). As of 2020, the system had 2.63 million, accounting for 5.1% of the entire population (9). According to previous literature, people with disabilities are more likely to have chronic diseases. For example, 84.3% of people with disabilities have chronic conditions, 1.8 times higher than those without disabilities (46.5%) in 2017 (10). In addition, people with disabilities are physically inactive, have a higher proportion of osteoporosis, and have an impaired quality of life compared to those without disabilities (11). While they are less likely to receive preventive screening services, e.g., cervical cancer screenings (11, 12) or gastric cancer screenings (13).

Meanwhile, they are more likely to use healthcare services; for example, they have a higher length of stay for inpatient care (14), and their healthcare expenditure is four times higher than persons without disabilities (5,375 thousand KRW vs. 1,298 thousand KRW) in 2017 (10).

Although people with disabilities spend more resources on their healthcare, they meet the problems of access to healthcare. According to the concept of “Patient Centered Access to Healthcare,” there are key three outcomes: reduction of unmet health care needs, avoidable hospitalization, and emergency department admission (15, 16). Increasing access to primary care services is known to be associated with an improvement of these three outcomes, complementarily (15). Among them, there are considerable problems in unmet health care needs and avoidable hospitalization among persons with disabilities. For example, persons with disabilities, such as those with brain and physical impairments, experience more unmet healthcare needs (17, 18), and those with intellectual and developmental disabilities are more likely to be hospitalized due to diabetes-related ambulatory care-sensitive conditions (19). As a result, they experience poorer health outcomes, e.g., a higher incidence of cardiovascular disease and higher mortality rates than those without disabilities (20).

Previous literature proposed a conceptual framework for understanding healthcare disparities experienced by individuals with disabilities, conceptualizing how a discrepancy between personal and environmental factors may cause limited access to healthcare and quality (21). For example, people with disabilities are socio-economically disadvantaged, have lower income and education levels, and have challenges participating in the workplace (22, 23). Their lower socioeconomic status intensifies the barriers to access to healthcare services, as they are vulnerable to cost-related difficulties, for example, a lack of health insurance or living near the poverty level without medical aid (18, 24–26).

In addition, they experience overlapped barriers because of disabilities, which are not usually observed or measured in surveys. Such barriers may include difficulties in public transportation (18, 27), lack of accommodations specific to their particular needs, and difficulties finding adequate medical professionals who welcome people with disabilities (28). There are also unmeasurable factors in personal characteristics, e.g., psychological distance to physician meetings (29) and health literacy problems (30), which determine the attitudes toward healthcare utilization or preventive behaviors. Therefore, we need to pay attention to the role of unobserved factors, which may cause healthcare disparities and can be further worsened by the presence of disability (31).

Despite the well-known disparities in access to healthcare by persons with disabilities, there is little evidence about the relative contribution of observed and unobserved characteristics that explain the gaps between persons with and without disabilities. The extent to which socioeconomic differences can explain the disparities between disabled and non-disabled individuals is unclear. Among the outcomes of access to healthcare, this study focused on reduction of avoidable hospitalization and unmet health care needs (15). To our knowledge, none has distinguished between explained disparities (using covariates) and unexplained disparities (as discrimination) among the total inequalities on “unmet healthcare needs” and “preventable hospitalization” respectively. Thus, the purpose of this study is to examine the inequality between people with and without disabilities on unmet healthcare needs and preventable hospitalization, and measuring the explained and unexplained disparities in the two dependent variables using the Oaxaca-Blinder approach.

## Methods

### Data Source and Participants

We obtained data from the Korea Health Panel (KHP), a nationally representative longitudinal study operated by the Korea Institute for Health and Social Affairs and the National Health Insurance Service of South Korea since 2008. Sample households were selected using a two-stage cluster method from the population census data of Statistics Korea. Surveys were conducted annually on all eligible household members using the computer-assisted personal interviewing technique. The KHP provides information on health conditions, unmet needs, healthcare utilization, socioeconomic characteristics, and demographic characteristics and has been used to analyze unmet needs and healthcare utilization (32, 33).

The KHP survey questions for defining the disability are as following: “Has (name of household member) been assessed for disability?”, and if a respondent answered “Yes, assessed as having a disability + registration,” the respondent has been defined as “persons with disability.” The disability registration system is operated by Ministry of Health and Welfare and Ministry of Patriots and Veterans Affairs, and the definition of persons with disabilities refer to persons who have been severely restricted in daily life or social life for a long time due to physical or mental disabilities: Among them, physical disability refers to major external body function disorders and internal organ disorders, and mental disability refers to a disability caused by a developmental disability or mental illness (34).

The baseline sample included 7,866 households and 24,616 household members in 2008, and about 2,500 households were added in 2013 to compensate for panel attrition (35). The sample included 6,821 households and 18,870 household members in 2016, 6,497 households and 17,453 household members in 2017, and 6,493 households and 17,160 household members in 2018.

This study used data from individuals aged 18 years or older from the 2016–2018 KHP. Our sample was 43,517, including 3,027 (7%) observations with disability and 40,490 (93%) observations without disabilities. The analytical observations for unmet needs were 43,512 because the dependent variable of unmet healthcare needs has a missing value for five observations - three observations without disability and two observations with disability. We received institutional review board exemptions from the Public Institutional Bioethics Committee designated by the Ministry of Health and Welfare (IRB No. P01-202107-22-021).

### Measures

#### Dependent Variables

Our analyses used unmet healthcare needs and preventable hospitalization as dependent variables. Unmet healthcare needs were measured as “yes” replies to the question, “Have you ever missed seeing a doctor or getting a medical checkup that was necessary during the last year?” referring to previous literature (32, 33).

We measured preventable hospitalization as hospitalization due to ambulatory care sensitive conditions (ACSC)-related diseases. ACSC has been used to assess the quality of primary and community care, that is, access to appropriate primary care that could prevent the need for admission to hospitals. In Korea, Jeong et al. proposed Korean ACSCs (36). They consulted a panel of Korean clinicians with the original US version of 22 ACSCs to identify Korean ACSCs and proposed a total of 13 conditions for the Korean ACSCs (KACSCs) (36). It includes grand mal status epilepticus, convulsions, severe ear, nose, and throat infections, chronic obstructive pulmonary diseases, asthma, congestive heart failure, hypertension, angina, cellulitis, diabetes, hypoglycemia, gastroenteritis, and kidney/urinary tract infections (36). While KHP provided three diagnoses related to hospitalization, we classified the cases where the primary disease was consistent with ACSC-related diseases as hospitalizations due to ACSC, referring to the previous study (36).

#### Explanatory Variables

We included demographic and socioeconomic factors and health conditions in the analysis. Demographic factors included age, age squared, and sex. Socioeconomic factors included the existence of a spouse (yes or no), household income (low, middle, or high), an education level (middle school, high school, or university or above), employment status (employed or unemployed), residence (metropolitan or rural areas), and healthcare coverage (National Health Insurance (NHI) or Medical aid). Income groups were categorized into three groups, lower than 50%, 50%–150%, and higher than 150% of the median of equivalized household income. The medical aid program is a public aid scheme to secure access to health services for the low-income population. Health conditions included having chronic diseases (0, 1, 2, or 3+) and year dummy variables (2016, 2017, 2018).

### Statistical Analysis

We performed a descriptive analysis of dependent and explanatory variables for people with and without disabilities. We examined differences in contributors to the incidence of unmet healthcare needs and preventable hospitalization for adults with or without disabilities, respectively, using Ordinary Least Square (OLS) methods, that is, the linear probability model (LPM), referring to previous studies (37, 38). When using the logit or probit model, the estimation in the Oaxaca-Blinder decomposition depends on reference groups. For binary outcomes, a convenient alternative might be to use the Oaxaca-Blinder approach with the linear probability model (39). Thus, we interpreted the results from the linear probability model while showing both results from LPM and the logit model. With LPM, we interpreted $\beta_{j}$ as the expected change in the probability of an event occurring due to a unit change in $X_{j}$ , holding all other variables constant. We tried to reduce the potential sources of bias by adjusting demographic, socioeconomic variables, and health conditions. Next, we used an Oaxaca-Blinder approach to decompose the observed differences in dependent variables by disability status into explained and unexplained components (40, 41). The explained component reflects part of the gap attributable to the group differences in the explanatory variables, such as demographic and socioeconomic factors. The unexplained component reflects the residual difference that cannot be accounted for by the explanatory variables. We examined the detailed decomposition of the explained component using the Oaxaca command (42) and conducted all analyses using STATA software, version 16.

$${\bar{Y}}^{W}-{\bar{Y}}^{WO}=\sum_{j}\beta_{j}^{W}{\bar{X}}_{j}^{W}-\sum_{j}\beta_{j}^{WO}{\bar{X}}_{j}^{WO}=\sum_{j}\beta_{j}^{W}\left({\bar{X}}_{j}^{W}-{\bar{X}}_{j}^{WO}\right)-\sum_{j}\left(\beta_{j}^{W}-\beta_{j}^{WO}\right){\bar{X}}_{j}^{WO}$$

Note: w: with disability, wo: without disability.

## Results

### General Description

Table 1 shows the descriptive characteristics of disability status. The proportion of unmet needs was 15.36% and 9.76% for persons with and without disabilities, respectively. The proportion of preventable hospitalizations was 1.82% and 0.61% for persons with and without disabilities, respectively. In addition, persons with disabilities had more disadvantaged characteristics, such as a higher proportion of older individuals, no spouses, low education levels, unemployment, low income, chronic illness, being Medical aid recipients, and living in small cities compared to those without disabilities. These differences in characteristics of persons without disabilities may explain the gaps in unmet healthcare needs and preventable hospitalization, which were 5.6% points and 1.21% points, respectively (Table 1).

**TABLE 1:** Distribution of dependent and explanatory variables for people with and without disability (Republic of Korea. 2016–2018).

		Without disability	With disability	*p*-value^a^
Dependent variables				
Unmet healthcare needs*		9.76%	15.36%	<0.001
Preventable hospitalization		0.61%	1.82%	<0.001
Explanatory variables				
Sex	Female	50.51%	44.11%	<0.001
	Male	49.49%	55.89%	
Age	Age (mean)	46.24	61.28	<0.001
	Age square (mean)	2424.20	4020.55	
Spouse	No	39.61%	44.13%	<0.001
	Yes	60.39%	55.87%	
Education level	Less than high school	30.22%	56.57%	<0.001
	High school and over	69.78%	43.43%	
Working status	Unemployed	36.12%	64.34%	<0.001
	Employed	63.88%	35.66%	
Income level	Low income	13.21%	41.62%	<0.001
	Middle income	64.36%	49.39%	
	High income	22.42%	8.99%	
Residence	Metropolitan	45.85%	37.27%	<0.001
	Rural areas	54.15%	62.73%	
Chronic disease	No chronic disease	47.02%	13.42%	<0.001
	One chronic disease	20.12%	16.23%	
	Two chronic diseases	11.61%	15.67%	
	Three or more chronic diseases	21.26%	54.69%	
Health care coverage	National Health Insurance	97.41%	79.36%	<0.001
	Medical aid	2.59%	20.64%	
year	2016	33.02%	32.15%	0.850
	2017	33.35%	33.79%	
	2018	33.63%	34.06%	
No. of observations		40,490	3,027	

^a^ Note: Chi-square for categorical variables and *t*-test for continuous variables; *Two persons with disability and three persons without disability who did not report unmet care needs were excluded.

### Unmet Healthcare Needs for People With and Without Disability

The first and second columns of Table 2 show the regression results on unmet healthcare needs. For persons without disabilities, the rate of unmet healthcare needs of male persons without disabilities was approximately 2.1% lower than that of female persons without disabilities. Compared to the no-spouse group, those with spouses had a lower rate of unmet healthcare needs, about 1.7%, and those employed had unmet healthcare needs at a rate of approximately 4.1% lower. When the income level is high, the rate of unmet healthcare needs also decreases. When the income level is in the middle or high class, the unmet healthcare experience rate is reduced compared to those with low income. The unmet healthcare experience rate of Medical aid beneficiaries was about 6.7% higher than that of NHI enrollees. For persons with disabilities, the unmet healthcare needs of male persons with disabilities were approximately 4.0% lower than that of female persons with disabilities. When the income level is middle or high, the rate of unmet healthcare needs also decreases. The coefficients of sex and income level were greater for persons with disabilities than those without disabilities (Table 2).

**TABLE 2:** Regressions on unmet healthcare needs for people with and without disability (Republic of Korea. 2016–2018).

	Linear probability model				Logit model			
	Without disability		With disability		Without disability		With disability	
	Coeff. (s.e.)	*p*-value	Coeff. (s.e.)	*p*-value	Coeff. (s.e.)	*p*-value	Coeff. (s.e.)	*p*-value
Male	−0.0211	***	−0.0404	*	−0.2447	***	−0.3290	*
(ref. = female)	(0.004)		(0.019)		(0.043)		(0.142)	
Age	0.0026	**	0.0075	*	0.0335	***	0.0656	*
	(0.001)		(0.003)		(0.010)		(0.029)	
Age square	−0.0000	*	−0.0001	*	−0.0002	*	−0.0005	*
	(0.000)		(0.000)		(0.000)		(0.000)	
With spouse	−0.0165	**	−0.0201		−0.1614	*	−0.1352	
(ref. = no)	(0.006)		(0.021)		(0.064)		(0.160)	
High school and over	0.0032		0.0246		0.0947		0.1997	
(ref. = less than high school)	(0.005)		(0.022)		(0.063)		(0.172)	
Employed	0.0412	***	−0.0321		0.4977	***	−0.2746	
(ref. = unemployed)	(0.004)		(0.020)		(0.052)		(0.170)	
Middle income	−0.0330	***	−0.0748	***	−0.3337	***	−0.5640	***
(ref. = low income)	(0.007)		(0.019)		(0.069)		(0.147)	
High income	−0.0509	***	−0.1082	***	−0.5667	***	−0.9356	**
(ref. = low income)	(0.008)		(0.028)		(0.091)		(0.310)	
Rural areas	−0.0025		0.0207		−0.0331		0.1730	
(ref. = metropolitan)	(0.005)		(0.020)		(0.055)		(0.162)	
One chronic disease	0.0226	***	−0.0262		0.2722	***	−0.2539	
(ref. = no)	(0.006)		(0.031)		(0.066)		(0.300)	
Two chronic diseases	0.0304	***	0.0182		0.3387	***	0.1178	
(ref. = no)	(0.008)		(0.036)		(0.083)		(0.312)	
Three or more chronic diseases	0.0271	***	−0.0120		0.2993	***	−0.1252	
(ref. = no)	(0.007)		(0.033)		(0.078)		(0.299)	
Medical aid	0.0673	***	0.0431		0.5729	***	0.2946	
(ref. = National Health Insurance)	(0.016)		(0.027)		(0.113)		(0.173)	
2017	0.0050		−0.0262		0.0606		−0.2125	
(ref. = 2016)	(0.004)		(0.020)		(0.053)		(0.156)	
2018	0.0128	**	−0.0175		0.1470	**	−0.1354	
(ref. = 2016)	(0.004)		(0.020)		(0.052)		(0.154)	
Constant	0.0274		0.0166		−3.3234	***	−3.0789	***
	(0.018)		(0.084)		(0.213)		(0.843)	
F	31.48	***	3.993	***				
No. of observations	40,487		3,025					

Note: Ref. = reference group. *p < 0.05, **p < 0.01, ***p < 0.001; The number of observations included all respondents who were 18 years or older. Custer standard errors were used.

Table 3 shows the results of decomposing the disabled–non-disabled gap using descriptive statistics and regression analysis results. The disabled–non-disabled disparities in unmet healthcare needs due to characteristic effects, that is, distributional effect, was 2.92% point, accounting for 52% of the total gap. If people with and without disabilities had the same characteristics, the unmet need for medical care for those without disabilities would have increased by about 2.92% from 9.76%. Detailed decomposition of the distributional effect showed that sex, age, working status, income level, Medical aid, and chronic disease significantly increased the disabled–non-disabled disparities. For example, low-income persons were more concentrated in the disabled group (Table 1) and were more likely to experience unmet needs (Table 2), which led to an increase in the disabled–non-disabled disparities (Table 3). However, sex and working status appeared to decrease the disabled–non-disabled disparities. That is, sex led to a decrease in disabled–non-disabled disparities (Table 3) since disabled groups had a higher rate of male participants (Table 1) who were less likely to experience unmet healthcare needs than female participants (Table 2). Results from logit models were similar to those from LPM.

**TABLE 3:** Decomposition of gap in unmet healthcare needs for people with and without disability (Republic of Korea. 2016–2018).

		Linear probability model			Logit model		
		Contribution	(s.e.)	*p*-value	Contribution	(s.e.)	*p*-value
Overall contribution to the gap	Total gap	0.0560	(0.010)	***	0.0560	(0.010)	***
	Distributional effect	0.0292	(0.004)	***	0.0265	(0.004)	***
	Coefficient effect	0.0267	(0.010)	**	0.0295	(0.010)	**
Detailed decomposition on distributional effect	Sex	−0.0014	(0.000)	**	−0.0015	(0.000)	**
	Age	0.0091	(0.003)	**	0.0118	(0.003)	***
	Spouse	0.0007	(0.000)		0.0007	(0.000)	
	Education level	−0.0008	(0.001)		−0.0023	(0.002)	
	Working status	−0.0116	(0.001)	***	−0.0132	(0.002)	***
	Income level	0.0118	(0.002)	***	0.0118	(0.002)	***
	Residency	−0.0002	(0.000)		−0.0003	(0.000)	
	Chronic disease	0.0094	(0.003)	***	0.0097	(0.003)	***
	Medical aid	0.0121	(0.003)	***	0.0097	(0.002)	***
	Year	0.0001	(0.000)		0.0001	(0.000)	

Note: *p < 0.05, **p < 0.01, ***p < 0.001.

### Preventable Hospitalization for People With and Without Disability

Table 4 shows the regression analysis results on preventable hospitalization for people with and without disabilities. As for persons without disabilities, the preventable hospitalization rate was higher in males than females by approximately 0.3%. Those with three or more chronic diseases were more likely to experience preventable hospitalization by 1.1%. For persons with disabilities, the preventable hospitalization rate of male persons with disabilities was higher than that of female persons with disabilities by approximately 1.5%. People not living in metropolitan and with three or more chronic diseases were more likely to experience preventable hospitalization by approximately 1.2% and 2.3%, respectively. The coefficients of sex and chronic disease were higher for persons with disabilities than those without disabilities (Table 4).

**TABLE 4:** Regressions on preventable hospitalization for people with and without disability (Republic of Korea. 2016–2018).

	Linear probability model				Logit model			
	Without disability		With disability		Without disability		With disability	
	Coeff. (s.e.)	*p*-value	Coeff. (s.e.)	*p*-value	Coeff. (s.e.)	*p*-value	Coeff. (s.e.)	*p*-value
Male	0.0030	**	0.0154	*	0.5743	***	0.8214	**
(ref. = female)	(0.001)		(0.007)		(0.160)		(0.296)	
Age	−0.0004		0.0008		0.0006		0.0559	
	(0.000)		(0.001)		(0.028)		(0.067)	
Age square	0.0000	*	−0.0000		0.0002		−0.0005	
	(0.000)		(0.000)		(0.000)		(0.001)	
With spouse	−0.0009		−0.0024		−0.2369		−0.1741	
(ref. = no)	(0.001)		(0.008)		(0.197)		(0.377)	
High school and over	0.0015		−0.0044		0.0570		−0.3614	
(ref. = less than high school)	(0.001)		(0.009)		(0.157)		(0.503)	
Employed	−0.0006		−0.0095		−0.1350		−0.7272	
(ref. = unemployed)	(0.001)		(0.005)		(0.165)		(0.406)	
Middle income	0.0019		−0.0066		0.2088		−0.3264	
(ref. = low income)	(0.002)		(0.005)		(0.180)		(0.298)	
High income	0.0001		−0.0079		−0.2124		−0.6490	
(ref. = low income)	(0.002)		(0.007)		(0.268)		(0.821)	
Rural areas	−0.0001		0.0117	*	−0.0156		0.7442	*
(ref. = metropolitan)	(0.001)		(0.006)		(0.143)		(0.356)	
One chronic disease	0.0019	*	−0.0005		0.6587	**	0.2384	
(ref. = no)	(0.001)		(0.005)		(0.237)		(0.958)	
Two chronic diseases	0.0006		−0.0033		0.4817		−0.3834	
(ref. = no)	(0.001)		(0.005)		(0.329)		(1.038)	
Three or more chronic diseases	0.0106	***	0.0229	*	1.5977	***	1.7688	*
(ref. = no)	(0.002)		(0.009)		(0.252)		(0.856)	
Medical aid	0.0082		0.0172		0.5696	*	0.6033	*
(ref. = National Health Insurance)	(0.004)		(0.010)		(0.269)		(0.275)	
2017	−0.0006		−0.0066		−0.0975		−0.3816	
(ref. = 2016)	(0.001)		(0.006)		(0.152)		(0.297)	
2018	0.0002		−0.0072		0.0232		−0.3902	
(ref. = 2016)	(0.001)		(0.005)		(0.150)		(0.270)	
Constant	0.0060		−0.0184		−6.6906	***	−7.1880	**
	(0.004)		(0.039)		(0.718)		(2.259)	
F	8.22	***	2.34	**				
No. of observations	40,490		3,027		40,490		3,027	

Note: Ref. = reference group. *p < 0.05, **p < 0.01, ***p < 0.001; The number of observations included all respondents who were 18 years or older. Cluster standard errors were used.

Table 5 shows the results of decomposing the disabled–non-disabled gap using descriptive statistics and regression analysis results. The disabled–non-disabled disparities in preventable hospitalization due to characteristic effects, distributional effect, was 0.79% point, accounting for 65% of the total gap. In other words, if people with and without disabilities had the same characteristics, the preventable hospitalization of people without disabilities would have increased by about 0.79% from 0.61%. Detailed decomposition of the distributional effect showed that sex, age, and chronic disease significantly increased the disabled–non-disabled disparities (Table 5). For example, persons with chronic disease were more concentrated in the disabled group (Table 1), while they were more likely to experience preventable hospitalization (Table 4).

**TABLE 5:** Decomposition of gap in preventable hospitalization for people with and without disability (Republic of Korea. 2016–2018).

		Linear probability model			Logit model		
		Contribution	(s.e.)	*p*-value	Contribution	(s.e.)	*p*-value
Overall contribution to the gap	Total gap	0.0121	(0.003)	***	0.0121	(0.003)	***
	Distributional effect	0.0079	(0.001)	***	0.0087	(0.001)	***
	Coefficient effect	0.0042	(0.003)		0.0034	(0.003)	
Detailed decomposition on distributional effect	Sex	0.0002	(0.000)	*	0.0003	(0.000)	*
	Age	0.0032	(0.001)	***	0.0027	(0.001)	***
	Spouse	0.0000	(0.000)		0.0001	(0.000)	
	Education level	−0.0004	(0.000)		−0.0001	(0.000)	
	Working status	0.0002	(0.000)		0.0003	(0.000)	
	Income level	−0.0003	(0.000)		−0.0000	(0.000)	
	Residency	−0.0000	(0.000)		−0.0000	(0.000)	
	Chronic disease	0.0035	(0.001)	***	0.0046	(0.001)	***
	Medical aid	0.0015	(0.001)		0.0009	(0.000)	
	Year	−0.0000	(0.000)		−0.0000	(0.000)	

Note: *p < 0.05, **p < 0.01, ***p < 0.001.

## Discussion

To our knowledge, this is the first study to evaluate the explained disparities (as covariates) and unexplained disparities (as discrimination) about “unmet healthcare needs” and “preventable hospitalization” between persons with and without disabilities in South Korea. Overall, persons with disabilities experienced a higher rate of unmet healthcare needs (15.36% vs. 9.76%) and preventable hospitalization (1.82% vs. 0.61%). The decomposition results showed that different characteristics between persons with and without disabilities accounted for 48% and 35% of the total gap for unmet healthcare needs and preventable hospitalization, respectively. It means that more than half of the difference in unmet healthcare needs and preventable hospitalization between persons with and without disabilities were unexplained components, which are not explained by the observed differences using the explanatory variables. To ensure the reliability of the analysis results, we showed the both of LPM and logit models, and we interpreted the results from the LPM because the results were not different regardless of using LPM and the logit model.

The current study showed that the gap was 5.6% for unmet healthcare needs due to disability, and the explanatory variables explained 52% of the total gap. The detailed decomposition showed that the gap increased with income level and Medical aid. The proportion of low-income and Medical aid was much higher among persons with disabilities than those without disabilities. Low income and Medical aid may intensify the higher probability of experiencing unmet healthcare needs among persons with disabilities because even relatively small expenses can be catastrophic to poor households with members with disabilities (43). People with low family income and high healthcare needs due to a disability may experience high medical expenditure burdens (43, 44), which may reduce their visits to adequate healthcare services and increase the experience of unmet healthcare needs. The results were similar to those of a previous study that found socioeconomic status to be one of the main factors of healthcare disparities between persons with and without disabilities (21).

The detailed decomposition in the present study showed that the gap was decreased by sex and working status, while a previous study found that currently employed groups are less likely to receive necessary healthcare services due to “lack of time” (45), regardless of disability status. Our results showed that a higher proportion of working people among those without disabilities than those with disabilities reduced the disability-related gap.

Our finding showed that the unexplained component (coefficient effect) accounted for 48% of the gap. Diverse factors can cause the unexplained component of the gap in unmet healthcare needs, e.g., environmental barriers, such as health delivery system factors (such as the geographic location of services, transportation), support and relationships (such as caregivers and immediate family members), provider access factors (such as accessibility of buildings and equipment, availability of specialists) (21), and communication skills of healthcare providers for persons with disabilities (46). In addition, there are unobserved factors in psychological needs (29) and personal health literacy (30), which affect the attitudes to hospital visits or preventive treatment, that are not measurable using survey questionnaires.

When the dependent variable was “preventable hospitalizations,” the result showed that the gap was 1.21% between persons with and without disabilities, and the explanatory variables, such as sex, age, chronic disease, and Medical aid, explained 65% of the total gap. When considering the effect of age and chronic conditions, the average age of persons with disabilities was 61.3, which is about 15 years higher than those without disabilities, and chronic conditions were more prevalent among persons with disabilities than those without disabilities. That is, persons with disabilities are composed of an older population, and many have disability-related secondary or age-related chronic conditions (47). According to previous literature, the prevalence of hypertension is 2.7 times higher, diabetes is 2.8 times higher, and cerebrovascular disease is about five times higher than that of people without disabilities (10). Since these diseases are ACSC-related, the probability of preventable hospitalization could be higher among persons with disabilities.

When persons with disabilities live in rural areas, compared to those living in metropolitans, they are more likely to experience preventable hospitalization (Table 4). Our result reflects the accessibility problems in rural areas, as persons with disabilities may experience inadequate transportation and lower personal aid while at a higher risk of social exclusion (48, 49). Thus, the accumulated problems would have increased the probability of experiencing preventable hospitalization in rural areas.

The unexplained component (coefficient effect) accounted for 35% of the total gap between persons with and without disabilities for preventable hospitalization. The unobserved factors may be individual factors, such as secondary conditions or functional limitations, environmental factors, such as difficulties in finding a good quality of primary care, or the limited timeliness of care (12, 13, 21). Persons with disabilities may also be at increased risk of preventable hospitalization because they experience difficulties in the daily management of ACSC. In addition, the related information, including symptoms and prevention, is not available in accessible formats such as print materials in Braille, sign language interpretation, audio provision, or graphics (1). Meanwhile, there was no significant effect of unmet healthcare needs on preventable hospitalization, for people with and without disabilities (Supplementary Material).

In South Korea, the government introduced several policies to reduce the gap between persons with and without disabilities. First, the “Act on Guarantee of Right to Health and Access to Medical Services for Persons with Disabilities” was enacted in 2015 and implemented in December 2017 (50). The Act is to improve the health of persons with disabilities by providing for matters concerning support to guarantee the right to health, establish a healthcare system, and guarantee access to healthcare for persons with disabilities. Based on this Act, a “pilot program of primary care physician system for persons with disabilities” has been implemented to improve continuous and comprehensive care (51). Additionally, to improve general health checkups more safely and conveniently, “disability-friendly medical examination institutions” have been implemented since 2018 (51). The above programs may reduce the unexplainable components of healthcare access disparities. Thus, further studies are needed to evaluate the effect of these new policy efforts.

There were several limitations of this study. First, the original sample retention rate was 53.8% since the KHP started in 2008 (72.3% for the additional sampling of 2013) for the 2016–2018 data (35). It may be susceptible to response bias, reducing the representativeness of the results. Second, the proportion of persons with disability (6.95%) was higher than that of national statistics (4.9% of the adults aged 20 years or older and 5.1% of the whole population, which includes only the disability registration system of Ministry of Health and Welfare) (9) because our study included all persons with disability in disability registration system both of Ministry of Health and Welfare and Ministry of Patriots and Veterans Affairs. Additionally, we did not distinguish the types and severity of the disability, as the sample size was insufficient to analyze the differences by disability type. Further studies need to consider the disability characteristics and the difference among persons with disability.

### Conclusion

This study compared unmet healthcare needs and preventable hospitalizations between persons with and without disabilities and divided the gap into explained and unexplainable components. The unmet healthcare needs and preventable hospitalizations were 5.6% and 1.21% higher in people with than without disabilities, respectively, of which 48% and 35% were due to characteristics that observed variables could not explain. Individual and environmental characteristics such as physical accessibility, having a caregiver to accompany them to a hospital, lack of appropriate primary care services, and invisible discrimination can be the possible components of the gap. This study is meaningful in showing the impact of invisible factors and the explainable personal characteristics in our society that causes the gaps in unmet healthcare needs and preventable hospitalization between persons with and without disabilities.
