# Supplementary material for: Decomposing Disability Inequality in Unmet Healthcare Needs and Preventable Hospitalizations: An Analysis of the Korea Health Panel
Source: Int J Public Health. 2023 Feb 28;68:1605312. doi: 10.3389/ijph.2023.1605312 (PMC10011105; doi:10.3389/ijph.2023.1605312)
Supplement: Supplementary file 1 [file DataSheet1.docx]

**Supplementary tables**

We examined differences in contributors to the incidence of preventable hospitalization for adults with or without disabilities using Ordinary Least Square (OLS) and logit models, including unmet needs as an explanatory variable. The results were similar to the models that did not include unmet healthcare needs as an explanatory variable. In addition, unmet needs were not significant contributors to the use of the incidence of preventable hospitalization.

Table S1. Regressions on preventable hospitalization for people with and without disability, including unmet healthcare needs

|  | Linear Probability Model | | | | Logit Model | | | |
| --- | --- | --- | --- | --- | --- | --- | --- | --- |
|  | Without disability | | With disability | | Without disability | | With disability | |
|  | Coeff.  (s.e.) | p-value | Coeff.  (s.e.) | p-value | Coeff.  (s.e.) | p-value | Coeff.  (s.e.) | p-value |
| Male | 0.0030 | *** | 0.0155 | * | 0.5814 | *** | 0.8285 | ** |
| (ref.=female) | (0.001) |  | (0.007) |  | (0.158) |  | (0.289) |  |
| Age | -0.0004 |  | 0.0008 |  | 0.0002 |  | 0.0546 |  |
|  | (0.000) |  | (0.001) |  | (0.029) |  | (0.068) |  |
| Age square | 0.0000 | * | -0.0000 |  | 0.0002 |  | -0.0005 |  |
|  | (0.000) |  | (0.000) |  | (0.000) |  | (0.001) |  |
| With spouse | -0.0009 |  | -0.0023 |  | -0.2296 |  | -0.1701 |  |
| (ref.=no) | (0.001) |  | (0.008) |  | (0.198) |  | (0.379) |  |
| High school and over | 0.0015 |  | -0.0044 |  | 0.0549 |  | -0.366 |  |
| (ref.=less than high school) | (0.001) |  | (0.009) |  | (0.157) |  | (0.511) |  |
| Employed | -0.0007 |  | -0.0095 |  | -0.1449 |  | -0.7237 |  |
| (ref.=unemployed) | (0.001) |  | (0.005) |  | (0.164) |  | (0.412) |  |
| Middle income | 0.0019 |  | -0.0065 |  | 0.2198 |  | -0.319 |  |
| (ref.=low income) | (0.002) |  | (0.006) |  | (0.182) |  | (0.307) |  |
| High income | 0.0002 |  | -0.0077 |  | -0.1975 |  | -0.6366 |  |
| (ref.=low income) | (0.002) |  | (0.007) |  | (0.271) |  | (0.823) |  |
| Rural areas | -0.0001 |  | 0.0116 | * | -0.0166 |  | 0.743 | * |
| (ref.=metropolitan) | (0.001) |  | (0.006) |  | (0.143) |  | (0.357) |  |
| One chronic disease | 0.0019 | * | -0.0004 |  | 0.6529 | ** | 0.2368 |  |
| (ref.=no) | (0.001) |  | (0.005) |  | (0.238) |  | (0.957) |  |
| Two chronic disease | 0.001 |  | -0.0033 |  | 0.4737 |  | -0.3872 |  |
| (ref.=no) | (0.001) |  | (0.005) |  | (0.331) |  | (1.039) |  |
| Three+ chronic disease | 0.0105 | *** | 0.023 | * | 1.5922 | *** | 1.7693 | * |
| (ref.=no) | (0.002) |  | (0.009) |  | (0.252) |  | (0.856) |  |
| Medical aid | 0.0081 |  | 0.0172 |  | 0.5465 | * | 0.6019 | * |
| (ref.=National Health Insurance) | (0.004) |  | (0.010) |  | (0.272) |  | (0.281) |  |
| Unmet healthcare needs | 0.0019 |  | 0.0018 |  | 0.2229 |  | 0.0861 |  |
| (ref.=no) | (0.002) |  | (0.009) |  | (0.199) |  | (0.393) |  |
| 2017 | -0.0006 |  | -0.0065 |  | -0.0969 |  | -0.3772 |  |
| (ref.=2016) | (0.001) |  | (0.006) |  | (0.152) |  | (0.297) |  |
| 2018 | 0.0001 |  | -0.0072 |  | 0.0205 |  | -0.3898 |  |
| (ref.=2016) | (0.001) |  | (0.005) |  | (0.150) |  | (0.269) |  |
| Constant | 0.006 |  | -0.0184 |  | -6.7057 | *** | -7.1757 | ** |
|  | (0.004) |  | (0.039) |  | (0.716) |  | (2.261) |  |
| F | 7.98 | ** | 2.33 | * |  |  |  |  |
| No. of observations | 40,487 |  | 3,025 |  | 40,490 |  | 3,027 |  |

*Note:* Ref. = reference group. * p<0.05. ** p<0.01, *** p<0.001; The number of observations included all respondents who were 18 years or older. Cluster standard errors were used

Table S2. Decomposition of gap in preventable hospitalization for people with and without disability, including unmet healthcare needs

|  | | Linear Probability Model | | | Logit Model | | |
| --- | --- | --- | --- | --- | --- | --- | --- |
|  | | Contribution | (s.e.) | p-value | Contribution | (s.e.) | p-value |
| Overall contribution to the gap | Total gap | 0.0121 | (0.003) | *** | 0.0121 | (0.003) | *** |
|  | Distributional effect | 0.0080 | (0.001) | *** | 0.0088 | (0.001) | *** |
|  | Coefficient effect | 0.0042 | (0.003) |  | 0.0033 | (0.003) |  |
| Detailed decomposition on distributional effect | Sex | 0.0002 | (0.000) | * | 0.0003 | (0.000) | * |
|  | Age | 0.0032 | (0.001) | *** | 0.0026 | (0.001) | *** |
|  | Spouse | 0.0000 | (0.000) |  | 0.0001 | (0.000) |  |
|  | Education level | -0.0004 | (0.000) |  | -0.0001 | (0.000) |  |
|  | Working status | 0.0002 | (0.000) |  | 0.0004 | (0.000) |  |
|  | Income level | -0.0003 | (0.000) |  | -0.0001 | (0.000) |  |
|  | Residency | 0.0000 | (0.000) |  | -0.0000 | (0.000) |  |
|  | Chronic disease | 0.0035 | (0.001) | *** | 0.0046 | (0.001) | *** |
|  | Medical aid | 0.0015 | (0.001) |  | 0.0009 | (0.000) |  |
|  | Unmet healthcare needs | 0.0001 | (0.000) |  | 0.0001 | (0.000) |  |
|  | Year | 0.0000 | (0.000) |  | -0.0000 | (0.000) |  |

*Note:* * p<0.05. ** p<0.01, *** p<0.001
